# Barriers and Facilitators to Telehealth in Underserved Rural Communities: A Mixed‐Methods Formative Evaluation

**DOI:** 10.1111/hex.70747

**Published:** 2026-06-26

**Authors:** Julia Terhune, Bree Holtz, Sabrina Ford, Katharine M. Mitchell, Charlie Hornbogen, Kelly A. Hirko

**Affiliations:** ^1^ Department of Advertising and Public Relations, College of Communication Arts & Sciences Michigan State University East Lansing Michigan USA; ^2^ Department of Obstetrics, Gynecology & Reproductive Biology, College of Human Medicine Michigan State University East Lansing Michigan USA; ^3^ Institute for Health Policy, College of Human Medicine Michigan State University East Lansing Michigan USA; ^4^ Bronson Healthcare Group Kalamazoo Michigan USA; ^5^ Department of Family Medicine, College of Human Medicine Michigan State University East Lansing Michigan USA; ^6^ Department of Epidemiology and Biostatistics, College of Human Medicine, Traverse City Campus Michigan State University Traverse City Michigan USA

**Keywords:** access, rural, telehealth, underserved

## Abstract

**Background:**

Telehealth can mitigate healthcare access barriers that contribute to persistent rural health inequities. However, a limited understanding of telehealth barriers and preferences hinders the implementation of effective programs in rural communities. We conducted a formative evaluation to identify barriers and facilitators to telehealth adoption in an underserved rural setting.

**Methods:**

We used a mixed‐methods design including surveys and in‐depth semi‐structured interviews with rural community stakeholders. The survey assessed telehealth utilisation, perceptions, technology access and barriers using Likert‐type scales. Survey data were analysed descriptively, and interview data were examined thematically across the Unified Theory of Acceptance and Use of Technology (UTAUT) constructs.

**Findings:**

Survey respondents (*n* = 59) were primarily female (73.2%), and White (73.2%), with a mean age of 61 years. Over half (51.8%) reported annual incomes below $20,000, and 18.6% lacked home internet access. While 59.3% had heard of telehealth, only 42.4% had used it. Among users, 61.9% reported satisfaction, and 71.4% agreed or strongly agreed that communication with providers was adequate. Key barriers included limited internet connectivity (72.8%), technology challenges (66.6%) and lack of knowledge about accessing telehealth (62.5%). Interview participants (*n* = 8) emphasised that trust and comfort with providers were central to acceptance and highlighted telehealth's convenience in reducing transportation barriers.

**Conclusion:**

In this study population, telehealth was generally acceptable, with trust and comfort with the provider driving acceptance. Barriers were primarily related to connectivity, infrastructure and digital literacy. Consistent with larger studies, these findings support investments in broadband access, digital literacy, technical support and building patient confidence in virtual care.

**Patient or Public Contribution:**

Members of the public were involved in the design, recruitment and dissemination phases of this study. We partnered with local community organisations serving rural populations to inform recruitment strategies and to distribute surveys to community members with lived experience of healthcare access barriers. Community stakeholders also participated directly in the research through survey completion and in‐depth interviews, providing critical insights into telehealth use, barriers and preferences. While the public was not involved in the initial study design or data analysis, their perspectives shaped the interpretation of findings, particularly regarding trust, technology use and access challenges. To support dissemination and ensure accessibility of results, we developed a one‐page summary of findings that was shared with participating community partner sites for distribution back to the communities involved.

## 1 Introduction

Telehealth refers to the delivery of health care and health communication through digital tools such as video conferencing, email or other digital media. Telehealth can improve access to care in rural areas by reducing transportation and travel‐time barriers and expanding the capacity of local clinicians to deliver high‐quality care [1, 2, 3]. Rural communities are well‐positioned to benefit from telehealth, as residents are often geographically isolated from healthcare services, face significant health professional workforce shortages, and experience poor health outcomes as a result [4]. Programs such as the Extension for Community Healthcare Outcomes (Project ECHO) illustrate the impact of telehealth in rural settings by connecting specialists with community clinicians to expand capacity for speciality care in underserved areas [5]. Similarly, telehealth‐supported hypertension monitoring programs have resulted in improved blood pressure control for rural populations in prior studies [6, 7]. Collectively, these findings suggest that telehealth can be effectively utilised by rural populations and meaningfully improve health outcomes.

Advances in telecommunications, expanded telehealth platform access, and increased training and adoption have further enabled rural residents to engage in remote care [6, 7]. Despite these advances, telehealth adoption remains disproportionately lower in rural than urban communities [2, 3, 8]. Lower telehealth adoption rates in rural populations may be driven by limited broadband access, fewer connectivity options, lower digital literacy and lower ownership of digital devices in rural areas [9, 10]. Indeed, the United States Department of Agriculture (USDA) reports that 14% of rural households lack internet access, compared to only 3% of urban households [11]. The lack of high‐speed internet access in rural areas is compounded by the high cost of broadband internet in rural regions [12]. These structural barriers intersect with higher proportions of older adults, adults living with chronic illness, individuals living in poverty, and those with lower educational attainment—populations that consistently demonstrate lower digital literacy [13]. Taken together, infrastructural and socio‐demographic factors suggest that although telehealth may be more challenging to implement in rural areas, its adoption could yield substantial benefits for rural populations.

Digital literacy is commonly defined as the ability to use information and communication technologies to find, evaluate, create and communicate information, requiring both cognitive and technical skills. Broader definitions also emphasise the influence of social and cultural context on digital engagement [14]. National data indicate that low digital literacy is more prevalent among adults aged 65 years and older, individuals with a high school education degree or less, and those not participating in the labour force [15]. These characteristics are more common in rural areas, where 85% of ‘older‐age counties’ are located, educational attainment is lower, and labour force participation lags behind urban communities [16, 17].

While structural barriers to telehealth use are well documented, less is known about how rural populations perceive telehealth barriers and benefits. Indeed, telehealth research often under‐represents rural communities, low‐income populations and older adults with limited digital literacy. This knowledge gap limits the implementation of acceptable and effective telehealth programs in rural communities, where geographic dispersion and limited healthcare access heighten the need for virtual care. To inform the implementation of more accessible and acceptable telehealth interventions, we conducted a formative evaluation to identify barriers and facilitators to telehealth and inform actionable equity‐informed guidance for telehealth program implementation in resource‐constrained rural settings.

## 2 Methods

We conducted a mixed‐methods formative evaluation assessing telehealth utilisation and perceived facilitators and barriers to telemedicine use among community members recruited through partnerships in rural Northern Michigan. Surveys were distributed in the following regions: Benzie County, Lake County and Beaver Island (in Charlevoix County), all of which were classified as rural using the 2013 USDA Rural‐Urban Continuum Codes and considered underserved based on designation by the Health Resources and Services Administration (HRSA) as low‐income population Health Professional Shortage Areas (HPSAs). Details of the study methodology and recruitment are reported in a prior publication [8]. Briefly, survey packets were distributed between March and September 2020 at two food pantries and one island‐based health centre, with facility staff serving as primary survey distributors. Adults aged 18 years or older were asked to complete the paper surveys and return them by mail using the provided self‐addressed stamped envelopes. Survey packets included a welcome letter, the paper survey, a self‐addressed stamped return envelope, an invitation to participate in a voluntary follow‐up phone interview, and a US $2 bill as an incentive to return the survey [8]. Paper surveys were used to accommodate limited internet access in these rural communities. Consistent with the Michigan State University Institutional Review Board‐approved protocol, consent was implied through participants' voluntary completion and return of the survey. The survey asked respondents to provide contact information if they were willing to participate in a follow‐up interview after completing the survey. Participants interested in the follow‐up interview returned the contact form with the study survey in the self‐addressed stamped envelope.

### 2.1 Data Collection

The paper survey in this study was adapted from instruments used in prior studies examining telemedicine use, perceptions and technology access [8, 18] and was informed by the Technology Acceptance Model [19] and the Unified Theory of Acceptance and Use of Technology (UTAUT) Model [20], widely used frameworks for understanding factors that influence technology adoption and use. The UTAUT framework proposes that individuals' acceptance of technology is shaped by four key constructs: performance expectancy (the perceived usefulness of the technology), effort expectancy (the perceived ease of use), social influence (the extent to which important others perceive technology use) and facilitating conditions (the availability of resources and support to enable use) [20]. The survey assessed respondents' prior telemedicine use, perceptions of telemedicine using a Likert‐type response scale (1 = strongly agree to 5 = strongly disagree), socio‐demographic factors, insurance and employment status, self‐reported health status, technology access, and primary care provider status.

To contextualise the quantitative responses from the survey, we interviewed a subset of survey respondents who agreed to participate in a follow‐up interview. The interview guide was developed through referenced survey responses and informed by the UTAUT model. Telehealth perceptions and barriers were assessed across the following UTAUT constructs: performance expectancy, effort expectancy, social influence and facilitating conditions [20].

### 2.2 Statistical Analysis

Descriptive statistics were used to characterise the study population. The reliability of survey questions was reported using Cronbach's *α*. In addition, frequencies and percentages were evaluated for variables describing barriers to telehealth use from those who had not used telehealth and intentions to use telehealth from previous users. We summarised barriers to using telehealth from non‐users and reasons for using telehealth among those who have used telehealth, triangulating survey results with feedback from the qualitative interview data using the UTAUT framework as a guide.

## 3 Results

### 3.1 Participant Characteristics

As shown in Table 1, survey respondents (*n* = 59) were primarily female (73.2%), and White (73.2%), with a mean age of 61 years. Over half (51.8%) reported annual incomes below $20,000, and 18.6% reported no home internet access. While 59.3% had heard of telemedicine, only 42.4% had previously used it.

**Table 1:** Characteristics of rural community survey respondents (*n* = 59).

	Overall *N* (%)
**Age, years**	
≤ 39	5 (8.5%)
40–49	8 (13.6%)
50–59	8 (13.6%
60–69	19 (32.2%)
70–79	9 (15.3%)
≥ 80	4 (6.8%)
Prefer not to answer	6 (10.2%)
**Gender**	
Female	41 (73.2%)
Male	14 (25.0%)
Prefer not to answer	4 (1.8%)
**Race and ethnicity**	
White	41 (73.2%)
Black or African American	3 (6.1%)
American Indian, Alaska native, Native Hawaiian	3 (6.1%)
Missing	12 (20.3%)
**Household income, $USD**	
< $20,000	29 (51.8%)
$20,000–$49,999	12 (21.4%)
$50,000–$99,999	7 (12.5%)
Missing	11 (18.6%)
**Educational attainment**	
< High school	3 (5.6%)
High school graduate	22 (40.7%)
Some college	18 (33.4%)
Bachelor's degree	4 (7.4%)
Graduate school	7 (13.0%)
**Employment status**	
Employed	10 (18.6%)
Unemployed	7 (13.0%)
Retired	20 (37.0%)
Unable to work/other	17 (31.5%)
**Health insurance**	
Private	5 (8.5%)
Medicaid	12 (20.3%)
Medicare	25 (42.4%)
Military	5 (8.5%)
None	4 (6.8%)
Missing	8 (13.6%)
**Internet access**	
Cell phone/mobile device	31 (52.5%)
Broadband internet	23 (39.0%)
Satellite internet	9 (15.3%)
Dial‐up internet	2 (3.4%)
No internet access	11 (18.6%)
Do not know	2 (3.4%)
**Study site**	
Beaver Island, Michigan	16 (27.1%)
Benzie County, Michigan	30 (50.8%)
Lake County, Michigan	13 (22.0%)
**Self‐reported health status**	
Excellent	7 (7.8%)
Very good	10 (16.9%)
Good	21 (35.6%)
Fair	9 (15.3%)
Poor	7 (11.9%)
Missing	5 (8.5%)
**Have a primary care provider**	
Yes	47 (79.7%)
No	8 (13.6%)
Missing	4 (6.8%)
**Heard about telemedicine previously**	
Yes	35 (59.3%)
No	17 (28.8%)
Missing	7 (11.9%)
**Used telemedicine previously**	
Yes	25 (42.4%)
No	16 (27.1%)
Missing	18 (30.5%)

### 3.2 Perceptions

Overall, those who had used telemedicine (*n* = 25) reported generally positive perceptions, with 61.9% reporting satisfaction with telemedicine visits, and 71.4% agreeing or strongly agreeing that communication with providers was adequate (Table 2). Most participants indicated that they were able to communicate adequately with their healthcare providers, felt comfortable that their healthcare provider was able to understand what was bothering them, were satisfied with the telehealth visit, rated the quality of care as excellent, and believed their healthcare provider genuinely seemed to care about them. Technical difficulties, including seeing or hearing the provider, were noted, with 28.6% reporting difficulty seeing the healthcare provider over the computer or mobile system. While many participants expressed a general preference for in‐person care (50% agreed or strongly agreed), they also recognised telehealth's value in reducing infection risk. Most telehealth users reported they would recommend telehealth to others (63.6% agreed or strongly agreed). Concerns about privacy and confidentiality were minimal, with 10%–15% expressing some degree of concern. Users largely described telehealth as convenient (66.6% agreed or strongly agreed), easy to use (80% agreed or strongly agreed), and made it easier to get medical care (50% agreed or strongly agreed). Nearly half of the participants agreed or strongly agreed that it was easy to access primary care appointments (15% strongly agreed and 30% agreed). Most participants indicated they would use telemedicine again (60% agreed or strongly agreed). Participants indicated moderate reliance on telehealth for situations such as after‐hours care or in situations when illness prevented travel to the clinic. However, concerns about insurance coverage were reported by 30% of respondents.

**Table 2:** Perceptions of telehealth among users (*N* = 25).

	Strongly agree	Agree	Neutral	Disagree	Strongly disagree
I was able to communicate adequately with the healthcare provider	52.4%	19.0%	19.0%	4.8%	4.8%
I was comfortable that the healthcare provider was able to understand what was bothering me	42.9%	23.8%	19.0%	9.5%	4.8%
I had difficulty hearing the healthcare provider over the computer/mobile system	4.8%	4.8%	19.0%	33.3%	38.1%
I had difficulty seeing the healthcare provider over the computer/mobile system	0%	28.6%	4.8%	14.3%	47.6%
I would have gotten better care if I had seen the healthcare provider in person	9.5%	14.3%	33.3%	19.0%	23.8%
Overall, I was very satisfied with telemedicine visits	47.6%	14.3%	23.8%	4.8%	9.5%
The healthcare provider dominated the conversation	5.0%	15.0%	30.0%	35.0%	15.0%
I am concerned that my primary care provider will not get my visit information	0.0%	15.0%	20.0%	30.0%	35.0%
The quality of care through telemedicine is excellent	19.0%	28.6%	38.1%	4.8%	9.5%
I am worried about the continuity of care (i.e., I don't see my same provider every time)	0.0%	5.0%	25.0%	30.0%	40.0%
The healthcare provider spent little time taking my medical history	5.0%	15.0%	20.0%	40.0%	20.0%
The healthcare provider who provided me care genuinely seemed to care about me	55.5%	15.0%	20.0%	5.0%	5.0%
I am worried about the accuracy of the information from the telemedicine healthcare provider	0.0%	14.3%	19.0%	42.9%	23.8%
The next time, I would prefer to see a healthcare provider in person, despite the possible inconvenience	30.0%	20.0%	20.0%	25.0%	5.0%
There was less communication with the provider (than I normally receive in person), using telemedicine	20.0%	10.0%	20.0%	25.0%	25.0%
I felt like my privacy was invaded during the telemedicine visit	5.0%	5.0%	15.0%	35.0%	40.0%
I am worried about the confidentiality of the private information being exchanged during the telemedicine visit	5.0%	10.0%	10.0%	25.0%	50.0%
I have used telemedicine because I didn't want to infect (cold, flu, etc.) other people in the waiting room	31.6%	15.8%	10.5%	36.8%	5.3%
I have used telemedicine because I didn't want to get infected in the waiting room (cold, flu, etc.)	36.8%	21.1%	10.5%	26.3%	5.3%
I would recommend telemedicine services to others	40.9%	22.7%	22.7%	4.5%	9.1%
I have used telemedicine because I didn't feel like my condition was too urgent	15.0%	20.0%	20.0%	30.0%	15.0%
Telemedicine made it easier to get medical care when I needed it	27.3%	22.7%	31.8%	9.1%	9.1%
It was convenient to receive care through telemedicine	44.4%	22.2%	16.7%	5.6%	11.1%
It was easy to arrange an appointment	40.0%	40.0%	5.0%	10.0%	5.0%
It is easy to get in to see my primary care provider	15.0%	30.0%	35.0%	10.0%	10.0%
I generally use telemedicine when my provider isn't open (e.g., after hours, holidays, etc.)	15.0%	5.0%	20.0%	35.0%	25.0%
I am concerned that my insurance will not cover my telemedicine visit	10.0%	20.0%	15.0%	30.0%	25.0%
If I had the opportunity, I would use telemedicine again	45.0%	15.0%	20.0%	0.0%	20.0%
I generally use telemedicine when I feel too sick to leave the house	15.0%	10.0%	20.0%	30.0%	25.0%

### 3.3 Barriers

As shown in Table 3, key barriers included limited internet connectivity (72.8%), technology challenges (66.6%), and lack of knowledge about accessing telemedicine services (62.5%). Non‐users of telehealth reported multiple barriers to utilising telehealth. Many participants believed they would receive better care in person (54.5% strongly agreed and 9.1% agreed) and were concerned about continuity of care (30% agreed) and communication quality (10% strongly agreed and 30% agreed). Most were neutral regarding confidentiality of information through telehealth (70% neutral) and whether primary care providers would receive their telehealth visit information (70% neutral). The preference for in‐person care was also reflected, with participants noting they lacked telehealth ‘know how’ and found in‐person visits easy to access. Some participants expressed a preference for walk‐in clinics over telehealth (40% agreed or strongly agreed), suggesting social norms may favour in‐person care. Lack of knowledge about how to access or locate telehealth services was common, with 62.5% agreeing or strongly agreeing they did not know how to get telehealth care and 12.5% uncertain. Some also perceived that telehealth visits may take longer than in‐person visits (20% agreed or strongly agreed). Barriers related to facilitating conditions were primarily related to aspects of technology and access to infrastructure. Over 70% of participants cited limited internet quality and 66.6% reported insufficient technological skills, while others lacked the necessary devices (30%) or were unsure if insurance would cover telehealth visits (63.7%).

**Table 3:** Barriers to telehealth among non‐users constructs (*N* = 16).

	Strongly agree	Agree	Neutral	Disagree	Strongly disagree
**I have not used telemedicine because…**					
I think I would get better care in person	54.5%	9.1%	27.3%	9.1%	0.0%
I would get better care if I see my provider in person	40.0%	10.0%	40.0%	10.0%	0.0%
I prefer going to walk‐in clinics	20.0%	20.0%	40.0%	10.0%	10.0%
I worry about the quality of communication with a provider using telemedicine	10.0%	30.0%	30.0%	30.0%	0.0%
I worry about the continuity of care (i.e., I don't see the same provider every time)	0.0%	30.0%	40.0%	30.0%	0.0%
I worry that the healthcare provider will not be sensitive to my needs	0.0%	30.0%	30.0%	40.0%	0.0%
I think it would take longer to have a visit over telemedicine than in person	10.0%	10.0%	40.0%	40.0%	0.0%
I am worried about the ability to communicate adequately with the healthcare provider	10.0%	10.0%	30.0%	20.0%	30.0%
I am concerned that my primary care provider would not get my visit information	0.0%	0.0%	70.0%	30.0%	0.0%
I worry about confidentiality of my private information being exchanged through telemedicine	0.0%	10.0%	70.0%	20.0%	0.0%
It is easy to get into my primary care provider	27.3%	27.%	36.4%	9.1%	0.0%
I don't have very good internet	36.4%	36.4%	18.2%	9.1%	0.0%
I am not technologically savvy enough to use telemedicine services	33.3%	33.3%	0%	22.2%	11.1%
I am unsure if my insurance covers these visits	27.3%	9.1%	27.3%	18.2%	18.2%
I don't have the technology needed for telemedicine visits	20.0%	10.0%	40.0%	30.0%	0.0%
I don't know how to get telemedicine care	12.5%	50.0%	12.5%	25.0%	0.0%
I don't know how to find telemedicine services	10.0%	20.0%	60.0%	10.0%	0.0%
					

Themes and relevant quotes from qualitative interviews are shown in Tables 4 and 5. Interview participants (*n* = 8) noted telehealth's convenience in reducing transportation barriers but emphasised limited internet access as a barrier to utilisation. Participants viewed telehealth as beneficial for reducing travel time and costs, particularly in geographically isolated settings. Many appreciated being able to connect from home and felt that provider communication remained personal and meaningful in the virtual setting. However, concerns remained about the inability to conduct physical examinations and the perception that certain services are better suited for in‐person care. Several participants expressed conditional openness to telehealth but maintained a preference for face‐to‐face visits for more complex medical care. Telehealth was frequently described as convenient, time‐saving and easy to schedule, with some participants indicating increased willingness to try telehealth after learning how simple it was to use. However, challenges emerged around navigating virtual visits, highlighting usability considerations. Trust and comfort with the provider appeared central to telehealth acceptance. Participants emphasised that feeling connected to their doctor, whether virtually or in‐person, shaped their overall experience. Structural and technological barriers were prominent, with participants reporting unreliable internet connections, dropped sessions requiring phone call backups, lack of consistent broadband access in island or rural settings, and limited access to devices (e.g., losing access to a school‐issued laptop). These infrastructure and equipment challenges often disrupted visits and represented significant barriers to telehealth use.

**Table 4:** Qualitative assessment of telehealth perceptions according to Unified Theory of Acceptance and Use of Technology constructs.

Performance expectancy		Social influence	
Reasons to use telehealth	Reasons to not use telehealth	Reasons to use telehealth	Reasons to not use telehealth
The healthcare provider who provided me care genuinely seemed to care about me I was able to communicate adequately with the healthcare provider Overall, I was very satisfied with telemedicine visits If I had the opportunity, I would use telemedicine again I was comfortable that the healthcare provider was able to understand what was bothering me. There was less communication with the provider (than I normally receive in person), using telemedicine The quality of care through telemedicine is excellent	I have not used telemedicine because I didn't feel like my condition was too urgent I would have gotten better care if I had seen the healthcare provider in person I had difficulty hearing the healthcare provider over the computer/mobile system The healthcare provider dominated the conversation The healthcare provider spent little time taking my medical history I had difficulty seeing the healthcare provider over the computer/mobile system I am concerned that my primary care provider will not get my visit information I am worried about the accuracy of the information from the telemedicine healthcare provider I am worried about the continuity of care (i.e., I don't see my same provider every time)	I would recommend telemedicine services to others I have used telemedicine because I didn't want to get infected in the waiting room (cold, flu, etc.) I have used telemedicine because I didn't want to infect (cold, flu, etc.) other people in the waiting room	I am worried about the confidentiality of the private information being exchanged through the telemedicine visit. I felt like my privacy was invaded during the telemedicine visit
**Representative quotes from interviews**			
*‘It really benefits me the fact that I don't have to fly off the island and that costs me a lot of money, but the one thing that is versus them not really senior person is being able to touch you. The doctor can't really check my daughter's vitals or do really anything like that. So, there's a big difference in that because you don't really get to touch or the actual feeling of being with that person’.*		*‘Probably the connections that they are have with the doctor. If they feel comfortable with their doctor, whether they talking to them over the phone or seeing them face‐to‐face’.*	

Effort expectancy		Facilitating conditions	
Reasons to use telehealth	Reasons to not use telehealth	Reasons to use telehealth	Reasons to not use telehealth
It was convenient to receive care through telemedicine It was easy to arrange an appointment The next time, I would prefer to see a healthcare provider in person, despite the possible inconvenience Telemedicine made it easier to get medical care when I needed it It is easy to get in to see my primary care provider	I generally use telemedicine when I feel too sick to leave the house		I am concerned that my insurance will not cover my telemedicine visit I generally use telemedicine when my provider isn't open (e.g., after hours, holidays, etc.)
**Representative quotes from the interviews**			
*‘For me, what I liked is that I didn't have to go through all the trouble of traveling, taking a whole day to go for a doctor's office visit and then sitting in a waiting room. Especially in the pandemic, of course, you had everybody else sitting there too. For me, that was extremely beneficial. I could just connect from home directly to the doctor's office and have my chat with him and what I am concerned about or not concerned about. I didn't feel that I was like it was just a monitor. I did still feel this was a personal connection’.*		*‘Look, I have to travel to town. It takes me a good 20 min anyway to get into town to go to the med center, and the doctor isn't there. And I could just stay at home and schedule an appointment and do it right from my home and not have to travel into town unless it was necessary’.*	

**Table 5:** Qualitative assessment of telemedicine barriers according to Unified Theory of Acceptance and Use of Technology constructs.

		Social influence	
Performance expectancy	Reasons to not use telehealth	Reasons to use telehealth	Reasons to not use telehealth
I think I would get better care in person I would get better care if I see my provider in person I prefer going to walk‐in clinics I worry about the quality of communication with a provider using telemedicine I worry about the continuity of care (i.e., I don't see the same provider every time) I worry that the healthcare provider will not be sensitive to my needs I think it would take longer to have a visit over telemedicine than in person I am worried about the ability to communicate adequately with the healthcare provider	I am concerned that my primary care provider would not get my visit information	I worry about confidentiality of my private information being exchanged through telemedicine	
**Representative quotes from the interviews**			
*‘I would expect with some things I just feel like it's better in person like a dermatology appointment. Well, I haven't tried it with my primary care physician, but maybe if it's just questions because usually I have an appointment and I go in and we discuss it face to face, but I'd be willing to try it now that I know it's easy’.*		*‘Yeah. She doesn't quite understand [telemedicine], being a kid that you really need to sit there so your doctor can see you at all times. That's the part that she doesn't understand because she wants to vanish out of the way of the camera and stuff’.*	

Effort expectancy		Facilitating conditions	
			
It is easy to get into my primary care provider		I don't have very good internet I am not technologically savvy enough to use telemedicine services I am unsure if my insurance covers these visits I don't have the technology needed for telemedicine visits I don't know how to get telemedicine care I don't know how to find telemedicine services	
**Representative quotes from the interviews**			
*‘They couldn't get it to work on their end. I couldn't get it to work on my end…. But I do live in a place where sometimes it's difficult to get a connection. I mean, it kind of comes and goes’.*		*‘Well, I did have a laptop through the school where I work, but because I'm not going back there next year I had to give back the laptop’.* *‘Well, since I do live on an island, we have had issues on my computer where they've had to call me because the connection wasn't working good, so then they would have to call me on the phone and we would just put it on speaker and do it that way. So sometimes the technology is not the best because you have where we don't have the internet up and running or something so then we have to use speakerphone’.*	

## 4 Discussion

This study provides context‐specific insights into telehealth perceptions among a sample of underserved, low‐income rural adults—a population often under‐represented in research yet likely to benefit most from improved access. Findings from this study population suggest generally favourable perceptions of telehealth's effectiveness, convenience and acceptability, particularly for overcoming barriers to care. Strong patient–provider relationships appeared to enhance comfort with telehealth use. However, participants also described concerns about continuity of care, insurance coverage and privacy. Among non‐users, commonly reported barriers included limited internet connectivity, inadequate technology and digital skills, and uncertainty about how to access telehealth services. Although these findings are not intended to be representative of the broader rural population, they suggest that despite positive attitudes towards telehealth, technological, infrastructural and informational gaps continue to impede telehealth use among underserved rural residents. These results support ongoing efforts to improve digital infrastructure, technical support and user education to expand telehealth access in rural communities.

In this study, rural community stakeholders who had used telehealth generally perceived telehealth as effective, reporting adequate communication, high satisfaction and trust in provider care. These findings align with prior studies, highlighting performance expectancy as a key determinant of telehealth adoption, particularly when patients feel their healthcare needs are adequately addressed [21, 22]. Moreover, telemedicine adoption has been shown to increase when technologies are perceived to improve health outcomes, save time and meet patient expectations [22]. However, concerns about continuity of care and preference for in‐person visits reported by some participants in this study mirror findings from other studies showing that perceived limitations in care quality and continuity of care can reduce telehealth uptake [23]. Technological difficulties reported in this rural study population are generally consistent with prior evidence suggesting that many rural participants lack the infrastructure necessary to use telehealth, limiting the perceived performance of these tools [24]. Our findings suggest that performance expectancy may be influenced more by perceived clinical effectiveness than by technical usability in populations with sufficient digital literacy and access to technology infrastructure. Overall, findings from this study population suggest that while strong performance expectancy supports telehealth use, addressing concerns about continuity of care remains important for broader adoption. Our findings related to digital literacy challenges in this rural population also underscore the importance of addressing structural barriers, particularly broadband access, and digital literacy, before telemedicine adoption can be meaningfully expanded in rural settings.

Participants in this study indicated preferences for in‐person care and walk‐in clinics, reflecting social norms favouring face‐to‐face visits in rural areas. At the same time, many recognised telehealth's value for reducing infection risk and were willing to recommend telehealth to others, which aligns with research showing that support from trusted sources and positive subjective norms can increase telehealth acceptance [25]. Interestingly, despite rural populations often exhibiting higher levels of medical mistrust [25], participants in this study reported privacy and confidentiality concerns less frequently than general population samples [26]. These factors are well‐documented barriers that influence perceptions of telehealth's social acceptability and trustworthiness in broader adoption research. These findings suggest the importance of continuing to address data security and interpersonal concerns to enhance telehealth adoption, particularly in rural areas where trust in healthcare is generally lower [25].

Participants in this study generally perceived telehealth as convenient, easy to use and effective for accessing care, consistent with studies demonstrating that perceived ease of use increases adoption and sustained engagement with telehealth [27]. Moderate reliance on telehealth for after‐hours care or when illness prevents travel also reflects the importance of contextual usability in telehealth implementation efforts across diverse settings. However, concerns about insurance coverage, lack of knowledge about accessing telehealth, and perceived longer visit times are consistent with barriers identified in other settings. Taken together, these findings highlight that effort expectancy alone may not overcome structural and informational obstacles. Indeed, facilitating conditions, including reliable internet access, digital literacy training and support, are essential to enable equitable telehealth adoption, particularly in underserved or rural settings. These results are consistent with prior studies of rural telemedicine use [1, 28, 29, 30] and further align with results from studies conducted in African settings, which similarly identified limited broadband access, unreliable connectivity and low digital literacy as key barriers to telehealth utilisation [31]. Collectively, these parallels suggest that such challenges may not be unique to rural US populations but may represent common structural constraints across geographically and economically underserved contexts.

Notably, interview participants emphasised that established patient–provider relationships foster comfort with telehealth, aligning with prior research showing that preexisting trust enhances patient satisfaction regardless of the care modality [32]. Overall, qualitative data from participant interviews suggested that telehealth was valued for improving access and convenience in rural and geographically isolated communities, but its effectiveness was influenced by perceived clinical limitations and substantial technological and infrastructure constraints.

This mixed‐methods formative evaluation has several strengths. Grounding the survey and interview guide in the UTAUT framework provided a strong theoretical basis for assessing telehealth perceptions and allowed for systematic examination of performance expectancy, social influence and facilitating conditions. The use of both quantitative surveys and qualitative interviews enabled triangulation of findings and contextual depth to better understand telehealth barriers and facilitators in underserved rural communities. A notable strength of this study was its community‐engaged approach. Community partnerships in this rural region facilitated survey development and data collection, ensuring that the assessment was relevant and that data collection procedures were feasible. Recruitment through trusted community partners (food pantries and an island‐based health centre) enhanced engagement with economically vulnerable and geographically isolated populations that are often under‐represented in telehealth research. Use of paper surveys helped mitigate digital access barriers and was appropriate given COVID‐19 restrictions at the time of data collection. Several limitations should be considered when interpreting these findings. First, the sample size was modest (*n* = 59) relative to the participating communities and drawn from three rural regions in Northern Michigan. As a result, the findings should not be considered representative of these communities as a whole or generalisable to other rural settings. Rural populations differ widely by geography, climate, demographics and infrastructure, and telemedicine adoption barriers may vary accordingly. In addition, recruitment occurred through community partners using a convenience sampling approach, which may introduce selection bias by preferentially reaching individuals already engaged with local organisations. Because the survey was disseminated within feasible workflows of community partner organisations, a formal response rate could not be calculated. The predominance of older women in our study population may limit the generalisability of findings to younger adults and men. Technology‐related barriers observed in this study may be particularly salient among older adults with lower digital literacy. However, older adults also represent a substantial proportion of rural residents and healthcare users, making their experiences important for understanding telehealth implementation needs in rural settings. Self‐reported measures are subject to recall bias, and the cross‐sectional design precludes causal inferences regarding determinants of telehealth use. Finally, while the UTAUT framework guided measurement, some constructs were assessed using a limited number of items, which may not fully capture the complexity of telehealth adoption behaviours.

Taken together, findings from this study suggest that telemedicine is viewed as acceptable and valuable by rural residents who can access it, with trust and comfort with providers central to its use. Participants emphasised that feeling connected to their doctor, whether virtually or in‐person, shaped their overall experience with telehealth. Within our study population, barriers to telehealth were more commonly related to infrastructure, connectivity and digital literacy barriers than concerns about care quality. However, participants' preferences for in‐person care suggest that improving connectivity alone may not fully address telehealth utilisation gaps in rural settings. While these findings should not be generalised to all rural populations, they are consistent with evidence from larger studies and highlight potential areas for intervention, including broadband expansion, digital literacy support, patient‐centred technical support, and efforts to strengthen confidence in the quality and effectiveness of virtual care.

## Author Contributions

**Julia Terhune:** writing – original draft, investigation. **Bree Holtz:** conceptualisation, funding acquisition, investigation, writing – review and editing, methodology. **Sabrina Ford:** conceptualisation, investigation, funding acquisition, writing – review and editing, methodology. **Katharine M. Mitchell:** writing – review and editing, methodology, project administration. **Charlie Hornbogen:** writing – review and editing, visualisation. **Kelly A. Hirko:** conceptualisation, investigation, funding acquisition, writing – original draft, writing – review and editing, supervision, formal analysis.

## Ethics Statement

Michigan State University's Institutional Review Board approved this study (STUDY00004682) and all data collection materials.

## Conflicts of Interest

The authors declare no conflicts of interest.

## Data Availability

The data that support the findings of this study are available from the corresponding author upon reasonable request.
